# A suggested vital function for *eIF‐5A* and *dhs* genes during murine malaria blood‐stage infection

**DOI:** 10.1002/2211-5463.12093

**Published:** 2016-06-23

**Authors:** David Kersting, Mirko Krüger, Julia M. Sattler, Ann‐Kristin Mueller, Annette Kaiser

**Affiliations:** ^1^ Institute for PharmacogeneticsMedical Research CentreUniversity Duisburg‐EssenGermany; ^2^ Parasitology UnitCentre for Infectious DiseasesUniversity Hospital HeidelbergGermany; ^3^ Centre for Infectious Diseases, Integrative ParasitologyUniversity Hospital HeidelbergGermany; ^4^ German Center for Infectious DiseasesHeidelbergGermany

**Keywords:** hypusine, malaria, *Plasmodium*, reverse genetics

## Abstract

The biological function of the post‐translational modification hypusine in the eukaryotic initiation factor 5A (EIF‐5A) in eukaryotes is still not understood. Hypusine is formed by two sequential enzymatic steps at a specific lysine residue in the precursor protein EIF‐5A. One important biological function of EIF‐5A which was recently identified is the translation of polyproline‐rich mRNA, suggesting its biological relevance in a variety of biological processes. Hypusinated eIF‐5A controls the proliferation of cancer cells and inflammatory processes in malaria. It was shown that pharmacological inhibition of the enzymes involved in this pathway, deoxyhypusine synthase (DHS) and the deoxyhypusine hydroxylase (DOHH), arrested the growth of malaria parasites. Down‐regulation of both the malarial *eIF‐5A* and *dhs* genes by *in vitro* and *in vivo* silencing led to decreased transcript levels and protein expression and, in turn, to low parasitemia, confirming a critical role of hypusination in eIF‐5A for proliferation in *Plasmodium*. To further investigate whether eIF‐5A and the activating enzyme DHS are essential for *Plasmodium* erythrocytic stages, targeted gene disruption was performed in the rodent malaria parasite *Plasmodium berghei*. Full disruption of both genes was not successful; instead parasites harboring the episome for *eIF‐5A* and *dhs* genes were obtained, suggesting that these genes may perform vital functions during the pathogenic blood cell stage. Next, a knock‐in strategy was pursued for both endogenous genes *eIF‐5A* and *dhs* from *P. berghei*. The latter resulted in viable recombinant parasites, strengthening the observation that they might be essential for proliferation during asexual development of the malaria parasite.

**Abbreviations**
CNS: central nervous system
DHS: deoxyhypusine synthase
DOHH: deoxyhypusine hydroxylase
eIF‐5A: eukaryotic initiation factor 5A
NO: nitric oxide
PTM: post‐translational modification

Despite advances in control and chemotherapy of malaria, a mosquito‐borne infectious disease caused by single‐celled *Plasmodium* parasites, the disease is still responsible for the death of approximately 600 000 people annually 1. The architecture of a malaria infection can only be explained by a network combining immunological, molecular, and metabolic pathways 2. Hitherto, only a few pathways like fatty acid biosynthesis 3, the biosynthesis of *p*‐aminobenzoic acid in the shikimate pathway 4 and vitamin B_6_ 5 turned out to be essential in particular developmental stages for the survival of *Plasmodium*. Previous results demonstrating that heme biosynthesis is essential for the malaria parasite in the erythrocytic stages were recently challenged by knock‐out parasite lines, lacking 5‐aminolevulinic acid synthase and/or ferrochelatase (FC) 6. These knock‐out parasites grew normally in blood‐stage culture and exhibited no changes in sensitivity to heme‐related antimalarial drugs 6 due to their expression in the pre‐erythrocytic liver stages.

The novel amino acid hypusine 7 is a post‐translational modification that only appears in a single small acidic protein in the eukaryotic initiation factor 5A (EIF‐5A). Within two subsequent enzymatic steps, a specific lysine residue is modified by the enzymes deoxyhypusine synthase (DHS, EC 2.5.1.46) and deoxyhypusine hydroxylase (DOHH, EC 1.14.99.29) (Fig. 1). Hypusination is strictly linked to the polyamine pathway. While in the first step, under DHS catalysis, an aminobutyl moiety from the triamine spermidine is transferred to the ε‐amino group of a specific lysine in eIF‐5A (Lys 50 in EIF‐5A), DOHH completes hypusine biosynthesis by hydroxylation of the side chain in 2‐position and thus activates eIF‐5A in the second step.

**Figure 1:**
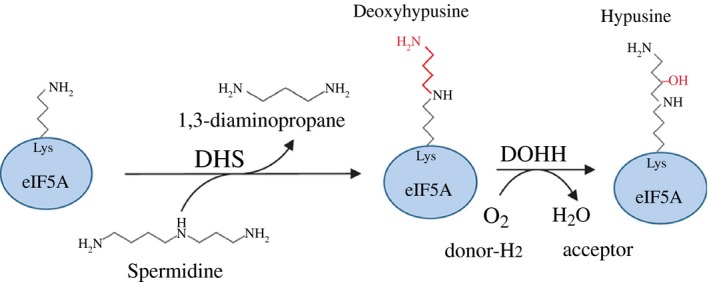
Schematic of the biosynthetic pathway of the post‐translational modification hypusine. Hypusine is formed within two subsequent steps. In the first step DHS transfers an aminopropyl moiety from the triamine spermidine to a specific lysine residue in the EIF‐5A precursor protein to form the deoxyhypusine intermediate. DOHH introduces a hydroxyl group into the side chain and thus completes hypusine formation. Targeting was performed for the eIF‐5A precursor protein and the DHS.

EIF‐5A has a central role in translation elongation 8 facilitating peptide bound formation during translation of polyproline‐containing proteins 9. Recent findings of a genome‐wide analysis of 35 representative organisms from six kingdoms of life, that is, archaebacteria, eubacteria, protista, fungi, plantae, and animalia showed that EIF‐5A‐dependent expression is strongly associated with specific biological processes like actin/cytoskeletal associated functions, RNA splicing/turnover, DNA binding/transcription, and cell signaling 10.

Over recent years, we have elucidated the hypusine pathway in a variety of human *Plasmodium* parasites and performed target evaluation of the enzymes DHS and DOHH, respectively 11, 12, 13, 14. Although there is the common opinion that the eIF‐5A‐modifying enzymes are highly conserved, this is, however, not true for the plasmodial enzymes in comparison to the human paralogues. The plasmodial DHS protein has very peculiar features containing stretches of asparagine and aspartate in the NAD‐binding site between the amino acid positions of serine 105 and aspartic acid 342 15 which do not appear in the human DHS. However, the most significant differences to the human ortholog appear in the spermidine‐binding site comprising the region between aspartic acid 243 and lysine 329 15. Moreover, DOHH from *Plasmodium falciparum* differs in the number of EZ‐like HEAT‐type repeats from its human counterpart 16, 17.

Hitherto, there is evidence that hypusine plays an important role in malaria infection, in particular in the blood stages. Pharmacological inhibition of either spermidine synthase or deoxyhypusine synthase 15 arrested parasitic growth *in vitro* in the erythrocytic stages suggesting that hypusine is involved in parasitic proliferation. Recent results 18 clearly demonstrated that the hypusine pathway in *Plasmodium* at least supports two different theories in malaria pathogenesis, that is, the sequestration theory and the inflammation hypothesis. One of the underlying mechanisms is the adherence of parasitized red blood cells to vascular endothelial cells by parasite specific proteins. Infected NMRI mice transfected with schizonts transgenic for *eIF‐5A* or *dhs* shRNA showed a 50% reduced parasitemia in comparison to the untransfected control within 2–9 days p.i. This may indicate the prevention of parasite invasion. Secondly, the inflammation hypothesis implies an inflammatory host response to the parasite in the central nervous system (CNS). Secretion of inflammatory cytokines like TNF‐α or IL1‐β leads to secretion of nitric oxide (NO) which kills the parasite. Our results demonstrated that NO concentration decreased in the blood stages of transgenic animals expressing either *eIF‐5A* or *dhs* shRNA. Moreover, it was shown that the impaired hypusination of parasitic eIF‐5A inhibited the nuclear export of the host iNos2 mRNA.

Till date, the essential nature of hypusine modification has been investigated in different multicellular eukaryotic organisms by deletion techniques of either of both of the activating enzymes alone. Whereas disruption of *eIF‐5A* or *dhs* genes is lethal in the fission yeast *S*. *cerevisiae* 17, a deleted DOHH null strain only grows at a slower rate than the wild‐type strain. In contrast, a deletion in the budding yeast *Schizosaccharomyces pombe* DOHH gene homolog *Mmd1* had a deleterious effect on mitochondrial morphology preventing microtubule stability and function 19. Most notably, the deletion affected E56 corresponding to E57 in the human homolog at one site of the strictly conserved HE residues for metal chelation.

EIF‐5A and DHS play an essential role in early embryonic development between E 3.5 and E 7.5 in mice. Heterozygous *eIF‐5A* and *dhs* mutants exhibited retarded growth of blastozyst development 20 due to belayed cell proliferation. By contrast, recent experiments demonstrated that hydroxylation of the deoxyhypusine side chain catalyzed by DOHH seems to be important only in a subset of multicellular organisms in a cell‐type specific manner. In the worms *Caenorhabditis elegans* (*C. elegans*) 21 and *Drosophila melanogaster* (*D. melanogaster*) 22, DOHH is essential. Recent results using gene targeting of the *dohh* gene in mice and *Caenorhabditis elegans*, demonstrated that DOHH activity is crucial for mammalian development, as well as for proliferation and oncogenic transformation of a fibroblast cell line 23. Thus, it seems likely, that eIF‐5A (Dhp50) and eIF‐5A (Hyp50) are not functionally interchangeable. Moreover, it was shown that the DOHH deletion has a strong impact on protein biosynthesis resulting in a 50% reduction 21 accompanied by a significant loss in eIF‐5A (Dhp50) which seems to be stabilized by hypusine. In addition, it was demonstrated that the knock‐out affected mostly those genes involved in cellular development, proliferation, and cancer 21.

Since conventional reverse genetic approaches are limited to study gene function in *Plasmodium*, we recently pursued an *in vivo* silencing approach based on RNAi of the *eIF‐5A* and *dhs* genes in the malaria parasite 24. Transfection of siRNA constructs into murine *Plasmodium* schizonts was performed which, in turn, were used for infection. Interestingly, mice transfected with eIF‐5A or DHS shRNA expression plasmids showed elevated parasitemia during the first 2 days after inoculation of transfected schizonts, followed by an intermittent decrease in parasitemia before succumbing death due to high parasitemia. These results were furthermore supported by RT‐PCR and western Blot analyses proving the downregulation of the EIF‐5A and DHS protein expression in gene‐specific shRNA‐transfected *Plasmodium berghei* ANKA schizonts.

Currently, gene targeting by homologous recombination is the most informative approach to study gene function in *Plasmodium*. Homologous recombination provides a versatile tool in manipulating the *Plasmodium* genome, that is, tens of kilobases can be spanned to produce large deletions and approximately 300 bp or less are sufficient for cross over formation 25, 26. Furthermore, the *Plasmodium* genome is haploid, contains mostly single copy genes and integrates exogenous DNA to ~ 100% by homologous recombination. Thus, for most genes a single recombination event is sufficient for generating a modified parasite clone. Crucially linear DNA is the preferred substrate for homologous recombination in *P. berghei*, a rodent malaria parasite. Transfected murine schizonts are then used for further infection of mice.

Since many examples showed a successful application of the replacement strategy in *Plasmodium,* we decided to investigate whether *eIF‐5A* and *dhs* genes might play an essential role in the proliferation of the parasite by loss of function in the rodent malaria parasite *P. berghei*. After successful transfection of parental schizont stages a selection based on pyrimethamine resistance of disrupted *eIF‐5A* and *dhs* genes was employed to further analyze whether the hypusine post‐translational modification (PTM) is essential for parasitic proliferation. To control for gene targeting at the desired locus and hence potential resistance to recombination events, we included an integration control (knock‐in strategy).

## Materials and methods

### Experimental animals and parasites

Female outbred NMRI mice were purchased from Janvier Labs, Saint‐Berthevin, France. All animal work was conducted in accordance with European regulations concerning FELASA category B and GV‐SOLAS standard guidelines and approved by the state authorities (Regierungspräsidium Karlsruhe). For the transfection studies we used *P. berghei* ANKA cl15cy1 (MRA‐871).

### *P. berghei eIF5‐A* and *DHS* gene targeting

Genomic DNA was extracted from mixed erythrocytic stages according to the Blood Amplification Kit protocol from Qiagen (Hilden, Germany) after infection of NMRI mice with *P. berghei* MRA‐871 ANKA strain cl15cy1. Two sets of oligonucleotides within the 5′ and 3′ UTR of the *eIF‐5A* and *dhs* genes were employed to perform the amplification from *P. berghei* genomic DNA for subsequent cloning into the targeting vector b3D 27. For the amplification of the eIF‐5A 5′UTR, primer *PbeIF5A*_5′ forward # 5′‐CCCAAGCTTATTTAGTAATGACACAAATCATAAAC‐3′ (35 bp) contained a *Hin*dIII restriction site (underlined) and primer *PbeIF5A*_*5′* reverse # 5′‐GGGGTACCAAAGAGTAATTCAAAATCATGTTTAAATA‐3′ (37 bp) a *Kpn*I (underlined) restriction site. Amplification of the 5′UTR of *dhs* was performed with primer *Pbdhs*_*5′* forward # 5′‐GGGGTACCCCACATATCAAAGGCCCACAAAATATCG‐3′ (36 bp) *(Kpn*I restriction site) and primer *PbDHS*_*5′* reverse # 5′‐CCCAAGCTTCCAATCAAAACATATGCATAG‐3′ (30 bp) (*Hin*dIII restriction site). The PCR reaction contained a volume of 25 μL: 2 μL genomic DNA (100 ng·μL^−1^) from *P. berghei* ANKA strain, 12.5 μL Master Mix (Ampliqon, Herlev, Denmark), and 4.5 μL water. Amplification was performed by PCR using a temperature profile of 95 °C 5 min, 95 °C 1 min, 50 °C for 1 min, 60 °C (for further specification 72 °C) for 2 min (30 cycles), 72 °C for 10 min for the 5′UTR of the *dhs* and the *eIF‐5A* genes, respectively. The obtained fragments of 481 bp for the 5′UTR of *eIF‐5A* and 565 bp for the 5′ UTR of the *dhs* gene were subcloned into the pSTBlue I Acceptor Vector (Merck, Darmstadt, Germany) and positive, recombinant clones obtained after transfection of Nova Blue competent cells were detected by PCR amplification. Restriction with *Kpn*I and *Hin*dIII was performed to subclone the 5′ UTR of the *eIF‐5A* and *dhs* genes into double digested *Kpn*I, *Hin*dIII b3D vector. For the cloning of the 3′UTR of the *dhs* and *eIF‐5A* genes, a similar strategy was pursued. Two sets of primer pairs were designed for amplification, that is, for 3′ UTR eIF‐5A primer *PbeIF5A*_3′ forward # 5′‐GGACTAGTGGTGATATTGCATATATGTGTC‐3′ (30 bp)‐3 (*Spe*I restriction site underlined) and primer *PbeIF5A*_*3′* reverse # 5′‐GCTCTAGAGGAAATACAATTGCCAAATAAATG‐3′ (32 bp) (*Xba*I restriction site underlined). The designed primer pair for the *dhs* gene fragment was primer *Pbdhs*_3′ forward # 5′‐GGACTAGTGGTATCGATTTAAAGGGAATAT‐3′ (30 bp) (*Spe*I restriction site underlined) and primer *Pbdhs*_3′ reverse # 5′‐GCTCTAGATGCTATATACTTTTCTTCGTAGCT‐3′ (32 bp) (*Xba*I restriction site underlined). Temperature profiles for the amplification of the 3′ UTR of the *eIF‐5A* gene and for the 3′ UTR of the *dhs* gene were: 95 °C 3 min, 95 °C 2 min, 58 °C 2 min, 60 °C 2 min (for further specification 72 °C; 30 cycles), 72 °C 10 min. The obtained fragments of 559 bp for the 3′ UTR of the *eIF‐5A* and 444 bp for the *dhs* gene were subcloned into pSTBlue I Acceptor vector (see Materials and methods within as already described for the 3′UTR constructs). Both fragments were further subcloned after *Xba*I*/Spe*I digestion into recombinant, digested *Xba*I b3D vector with the cloned 3′ UTR fragment. Since *Xba*I and *Spe*I produce the same compatible ends, *Xba*I digested b3D vector was dephosphorylated by calf intestine alkaline phosphatase and checked subsequently for the correct orientation of the insertion by *Xba*I*/Eco*RV digestion for the 3′ UTR of both genes. Both recombinant constructs were further used for transfection 28.

**Figure 2:**
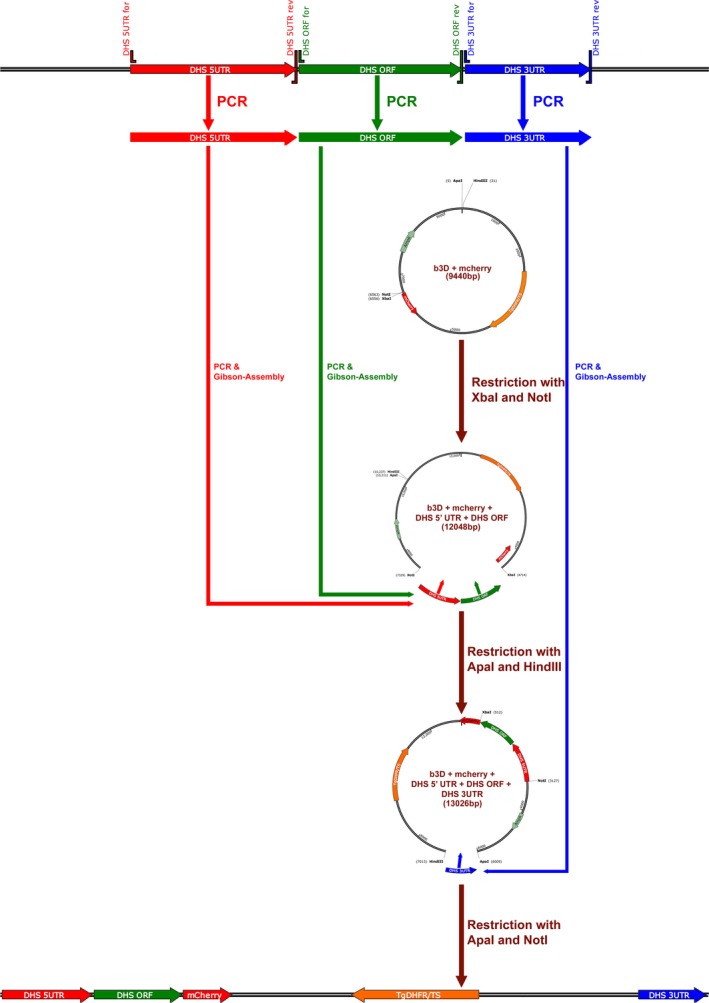
Schematic representation of the design of the knock‐in construct. Through two PCR amplification steps the 5′ UTR and the ORF of the *dhs* gene were amplified and assembled into the appropriately digested b3D^+^ Cherry vector. The amplified 3′UTR of the *dhs* gene was finally assembled into recombinant b3D+ mCherry vector 30.

For the control integration construct, that is, knock‐in of *eIF‐5A* and *dhs* genes a different strategy was used that comprised two subsequent steps. Genomic DNA from *P. berghei* MRA‐871 ANKA strain cl15cy1 was used as a template for PCR amplification of the 5′ UTR of the *dhs* gene employing the primer combination primer forward *Pbdhs* 5′UTR for # 5′‐CTCCACCGCGGTGGCGGCCGCTTATTTCATCAATTCCTTTAAAACAAC‐3′ (48 bp) (*Not*I restriction site underlined) and primer *Pbdhs* 5′UTR reverse # 5′‐CCATCCATTTTTTGATTTACATCTAGAATTAAACAGAG‐3′ (38 bp) (*Xba*I site underlined). For molecular cloning of the open reading frame (ORF) *dhs* primer *Pbdhs*ORF forward# 5′‐GTAAATCAAAAAATGGATGGGGTATTCAAAG‐3′ (31 bp) and *Pbdhs*ORF reverse # 5′‐CCTTGCTCACACTAGTTCTAGAATCACTTTTTTTCTCCTTTTCAC‐3′ (45 bp) were applied. To follow the Kozak rules, an additional base had to be introduced between the 5′UTR and the ORF of the *dhs* gene. The obtained two fragments for the *dhs* gene, that is, 5′UTR and ORF were purified and assembled to a fragment of 1317 bp according to a protocol from the Gibson Assembly Kit (Promega, Mannheim, Germany) 29 before it was inserted into *Not*I/*Xba*I double digested b3D+ mCherry vector 30 in the first step. Subsequent amplification of the 3′UTR of the *dhs* gene was performed with primers *Pbdhs* for 3′ UTR 5′‐ACCTGCAGGCAT GCAAGCTTAAGGATTAAGAAATAAAA ATATGATATAAGG–3′ (51 bp) (*Hin*dIII site underlined) and *Pbdhs* rev 3′ UTR 5′‐TGGTACCGGGCCCTTATTGCACCTCCTGAAAG‐3′ (32 bp) (*ApaI* site underlined) using an assembly reaction for cloning into recombinant b3D+ mCherry vector (containing the 5′ UTR *dhs* and ORF *dhs*) linearized with *Apa*I/*Hind*III. The detailed strategy is given in Fig. 2. The incubation mixture was heated at 50 °C for 45–50 min at 50 °C before it was transformed into highly competent *E. coli* DH5α cells. Recombinant clones were further characterized by restriction analysis.

A similar strategy was followed for the eIF‐5A integration construct. Amplification of the 5′ UTR of *eIF‐5A* and the ORF of *eIF‐5A* was performed with a set of two overlapping primer combinations before an assembly step followed. Although Kozak rules were fulfilled for the cloning of the *eIF‐5A* gene, an attempt to amplify the complete fragment, that is, the 5′ UTR and the ORF of eIF‐5A failed. Thus, amplification of the 5′UTR of the *eIF‐5A* gene and the ORF of the *eIF‐5A* gene were performed separately using the primer set *PbeIF‐5A* 5′UTR forward# 5′‐CTCCACCGCGGTGGCGGCCGCAAAATCCCAAATAATTTACCTGAC‐3′ (45 bp) (*No*tI restriction site underlined) and *PbeIF‐5A* 5′UTR reverse # 5′‐CCTTGCTCACACTAGTTCTAGATGATGACATTTCTTTAGCAGC‐3′(*Xba*I site underlined) (43 bp; and the assembly primer pair *PbeIF‐5A* ORF forward # 5′‐AAGATATAACAATTGGCTAGAAGTGTATTTATTTTAT AAAAAAG‐3′ (47 bp) and the primer pair PbeIF‐5A 5ORF reverse # 5′‐TAGCCAATTGTTATA TCTTTATGGGGCTGTTC‐3′ (32 bp). The PCR amplification reaction contained in a volume of 50 μL 300 ng genomic DNA from *P. berghei* ANKA strain, 250 pmol of each primer, 1 μL dNTP‐Mix (10 mm), 10 μL Q 5 buffer (Qiagen), and 0.5 U Taq polymerase (Qiagen).

For 3′ UTR amplification of the *eIF‐5A* gene, the primer combination *PbeIF‐5A for* 5′‐AAGATATAACAATTGGCTAGAAGTGTATTTATTTTATTATAAAAAAG‐3′ (47 bp) and *PbeIF‐5A* rev # 5′‐TAGCCAATTGTTATATCTTTATGGGCCCTGTTC‐3′ (32 bp) (*Apa*I site underlined) was applied. The obtained amplificate of 500 bp was finally assembled into recombinant b3D+ mCherry vector (containing the 5′ UTR *dhs* and ORF *dhs*) linearized with *Apa*I/*Hind*III. Recombinant clones were further characterized by restriction analysis.

Transfection of *P. berghei* schizonts was performed as previously described 31 using the Nucleofector Technology with linearized plasmids and gradient‐purified schizonts of the Pyrimethamine‐sensitive *P. berghei* strain ANKA generally comprising the three different main steps: (a) *in vitro* cultivation and enrichment *of P. berghei* schizonts, (b) transfection of recombinant DNA‐constructs containing drug‐selectable markers into schizonts, (c) infection of mice employing the transfected schizonts. Step 1: Blood from *P. berghei* ANKA infected mice at day 0 with a parasitemia of 5–15% was used to inoculate two mice until a parasitemia of 1–3% was reached. Blood was collected at day 4 from the infected animals to inoculate RPMI 1640 medium supplemented with FCS. The next day schizonts were enriched after Nycodenz‐PBS density centrifugation as a brownish interface. Step 2: Enriched schizonts were employed for electroporation of 8 μg of each DNA construct in two separate experiments. Step 3: Transfected schizonts were directly used to reinfect mice. Positive selection of stable integration continued for at least 9 days with pyrimethamine *ad libitum*. Resistant parasite populations were transferred to naïve mice for propagation and ultimate genotyping. Genotyping was performed by integration‐specific PCRs. For *dhs* integration, DHS Geno 5 forward primer# 5′‐GAAGTTGCAACCGATTTATTC‐3′ and DHS Geno 3 reverse primer # 5′‐GCATAAAAGGACCCATCATTATCC‐3′ were used. For *eIF‐5A* integration, PbeIF‐5A Geno 5 forward primer # 5′‐GTTGGGTACCCGAAAGTGTC‐3′ (20 bp) and PbeIF‐5A Geno 3 reverse primer # 5′‐GTACAAAAGTAGCTACTGTTATG‐3′‐ (23 bp) were employed. As control primers Tg forward # 5′‐CCC GCACGGACGAATCCAGATGG‐3′ and Tg reverse 5′‐CGCATTATATGAGTTCATTTTACACAATCC‐3′ which amplified a part of the coding region of the *Toxoplasma gondii* dihyrofolatreductase (DHFRS) were applied. Primer combinations of the genotyping primers (see above) with the primers for the respective wild‐type ORF of *eIF‐5A PbeIF‐5A for* ATGTCAGATCACGAAACTT (19 bp) and *Pbdhs* ORF for 5‐ATGGATGGGGTATTCAA‐3′ (17 bp) genes were combined for genotyping.

## Results

### Knock‐out studies of *eIF‐5A* and *dhs* genes during erythrocytic schizogony in *Plasmodium*

**Figure 3:**
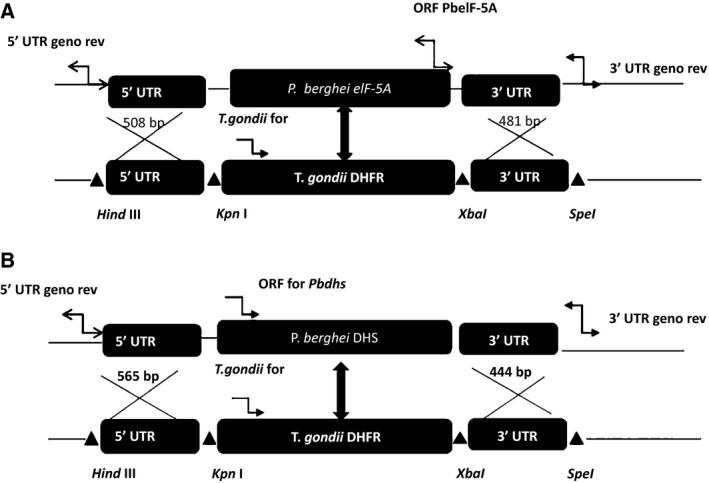
(A) Targeted gene disruption of the *eIF‐5A* gene in *Plasmodium berghei* ANKA strain by replacement strategy to generate a potential *PbeIF‐5A* knock‐out parasite. The PBA MRA‐871 ANKA strain cl15cy1 wild‐type strain *eIF5A l*ocus is targeted with a *Xba*I/*Hind*III linearized fragment containing the 5′UTR and the 3′UTR of the *eIF‐5A* gene and the *Toxoplasma gondii Dhfr*‐positive selectable marker. Upon a double crossover event the *PbeIF5A* ORF is replaced by *T. gondii Dhfr*‐positive selectable marker. Three replacement‐specific primer combinations marked with blue arrows were applied. (a) *T. gondii* ORF forward and 3′UTR *eIF‐5A* reverse primer (b) *T. gondii* ORF forward and *eIF‐5A* ORF reverse (c) *eIF‐5A* ORF forward and 3′UTR *eIF‐5A* reverse. (B) The replacement strategy for the generation of potential *Pbdhs p*arasites was similar to the targeted gene disruption for the *eIF‐5A* gene. The same primer combinations were employed except that *dhs* ORF primers forward and reverse, and 3′UTR *dhs* primer was used.

**Figure 4:**
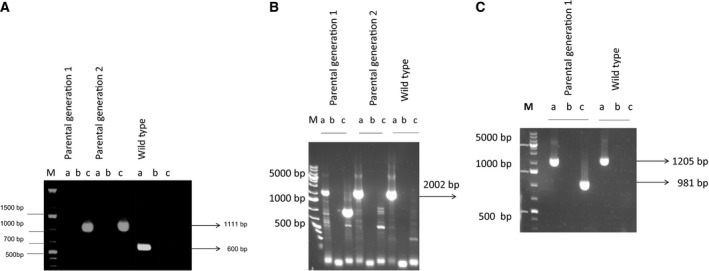
Replacement specific analysis of targeted gene disruption of the *eIF‐5A* and *dhs* genes: (A) Check of the predicted gene targeting of the 3′ UTR of the *eIF‐5A* gene by PCR analysis was performed using a set of gene‐specific primer combinations. PCR amplificates were analyzed on 1% agarose gels. M = 1 kb plus ladder (ThermoScientific, Darmstadt, Germany); 1a–c amplificates obtained from the transfected parental mouse 1; 2a–c amplificates detected from mouse 2 and the wild‐type *Plasmodium berghei* ANKA strain. Listed primer combination, which can give only a signal from the recombinant locus. (a) Primer # ORF *P. berghei eIF‐5A* forward + primer # *P. berghei eIF‐5A* genotype 3′ UTR reverse (b) Primer # *Toxoplasma gondii* forward + primer# *eIF‐5A* genotype 3′UTR reverse (c) Primer # *T. gondii* forward + primer# eIF‐5A ORF primer reverse. The results were obtained from two independent transfection experiments. (B) Replacement test primer combinations for the 3′UTR of the *dhs* gene by PCR analysis. 1a–c amplificates obtained from parental mouse 1; 2a–c amplificates detected from mouse 2 and 3a–c the wild‐type *P. berghei* ANKA strain. (C) 1a–c amplificates from the parental population represented by mouse 1, amplificates 2a–c from the wild‐type and the recombinant b3d^+^ mCherry vector 3a‐c. Replacement‐specific primer combinations: (a) Primer # ORF *dhs* forward + primer # *dhs* genotype 3′ UTR reverse; (b) Primer # *T. gondii* forward + primer *dhs* genotype 3′ UTR reverse; (c) Primer # *T. gondii* forward + primer# dhs ORF primer reverse.

To test whether *Plasmodium eIF‐5A* and *dhs* are essential genes for intraerythrocytic growth a reverse genetic approach in the rodent malaria model parasite *P. berghei* was used. To this end, a replacement strategy based on double homologous recombination was pursued to fully disrupt the *eIF‐5A* and *dhs* gene locus. We hence constructed gene respective targeting vectors, which upon a double cross over event during homologous recombination, would generate *eIF‐5A* and *dhs* knock‐out parasites (Fig. 3A,B upper panel and lower panel showing the strategies and recombination events), respectively. Targeting of the *eIF‐5A* and *dhs* gene wild‐type (WT) locus was performed with an *Xba*I and *Kpn*I‐linearized fragment containing the 5′UTR and 3′UTR of the *eIF‐5A* and *dhs* genes and the *Toxoplasma gondii* DHFR‐positive marker 29. Successful genetic replacement of both the *eIF‐5A* and *dhs* genes was checked in a PCR‐specific amplification with a set of three different primer combinations which were located outside the coding region of the dihydrofolate reductase gene in b3d backbone vector and outside the 3′ UTR of the *eIF‐5A* and *dhs* genes, respectively (Fig. 3A,B). Figure 4A depicts the results obtained in a PCR‐specific amplification for the 3′ integration of the *eIF‐5A* gene. Genomic DNA from two transfected mice representing the parental population was used as a template for amplification. Genotypical analysis for the 3′ integration of the *eIF‐5A* revealed an episomal integration of the recombinant b3D vector when the primer combination #*Tg for and eIF‐5A* 3′UTR *rev* were employed detecting a fragment of a size of 1111 bp (Fig. 4A, lanes parental generation 1c, 2c). Episomal integration in the two transfected mice was furthermore supported by the absence of a fragment for the 3′ integration with the expected size of 1366 bp when either the primer combination for the *eIF‐5A* ORF forward and genotype 3′ reverse primer (Fig. 4A, lanes parental generation 1a, 2a, wild‐type; 1330 bp) or the *T. gondii* dihydrofolate reductase forward (*Tg for*) and the *eIF‐5A* genotype 3′UTR (1366 bp) reverse primer were applied (Fig. 4A, lanes parental generation 1b, 2b). No fragment with an expected size of 1111 bp was obtained with the primer combination of *T. gondii dhfr* forward and Pb*eIF‐5A* Geno reverse when genomic DNA from the *P. berghei* wild‐type was used as a control (Fig. 4A, lane wild‐type c) while it was detectable in the two transfected mice (Fig. 4A, lanes parental generation 1c, 2c). The control experiment with primers *PbeIF‐5A* ORF forward and *PbeIF5A* ORF reverse detected the expected fragment of approximately 600 bp. These results were furthermore supported by genotyping of 5′ integration (Fig. 4C). When genotype primer 5′ UTR *PbeIF‐5A* forward and genotype primer *PbeIF‐5A* 3′ UTR reverse were combined the expected fragment of 2064 bp was detected in the wild‐type and in the parental population represented by gDNA obtained from mice 1 and 2 (Fig. 4C). However, the expected fragment of 5100 bp showing 5′ integration was absent in both parental lines. Although two independent parasite lines were generated and taken through two drug selection cycles, no integration into the parasite genome could be detected. Thus, it is conceivable that the *eIF‐5A* gene cannot be disrupted.

In parallel, genotyping was performed to investigate targeted gene disruption for the *dhs* gene locus. Figure 4B summarizes the results of the replacement‐specific PCR analysis. Again, genomic DNA from two transfected mice was employed. When the primer set combining Pb*dhs* ORF forward primer and *Pbdhs* 3′UTR geno rev for 3′ integration were applied, a signal of 2002 bp could be detected for both parental populations (Fig. 4B, lanes 1a, 2a) and the wild‐type Fig. 4B, lane 3a). However, the signal intensity for the parental mouse 1 (Fig. 4B, lane 1a) was weaker than for mouse 2 (Fig. 4B, lane 1a) and the wild‐type (Fig. 4B, lane wild‐type a). An expected fragment of 1003 bp was absent employing primer combinations *T. gondii* forward and *Pbdhs* primer 3′UTR reverse in transfected mouse 2 (Fig. 4C, lanes 1c). These results were furthermore confirmed by the absence of the fragment in the wild‐type control (Fig. 4C, wild‐type lane c) suggesting the presence of an episome in mouse 1 and no specific integration at the *dhs* gene locus, which has already been observed for the *eIF‐5A* locus. Moreover, genotypical analysis of the genomic DNA from the transfected, parental mouse 2 also pinpoints the occurrence of an episome. Episomal integration was further confirmed by using a combination of primers *Tg* forward primer and *Pbdhs* primer geno 3′ UTR rev (Fig. 4C, lanes 1c, 2c) demonstrating the absence of fragments with an estimated size of 1205 bp in both parental parasite strains (Fig. 4C, lanes 1c, 2c) and the wild‐type (Fig. 4C, lane 3c). Instead, artifacts of approximately 900 bp were detected (Fig. 4C, lanes 1c, 2c). To exclude any artifacts of fragments a different *Pbdhs* geno rev primer was designed resulting in the expected fragments of 981 bp in the parental population 1 (Fig. 4c, lane 1c) but not in the parental line 2 (Fig. 4c, lane 2c) In summary, there might be a lower percentage of episomal replicating parasites after transfection in the mixed population of the parental parasite line 2. Analysis of 5′ integration further strengthens our observation that the *dhs gene* might be essential for intra‐erythrocytic survival in blood stages of *Plasmodium*. A primer combination of *dhs* 5′ UTR geno forward primer (*dhs* geno for) and *dhs* 3′ UTR genotype reverse primer detected fragments of 3609 bp in the wild and the parental population instead of a fragment of 5809 bp in the case of full disruption.

### Plasmodial *dhs* and *eIF‐5A* knock‐in results in viable recombinant parasites

**Figure 5:**
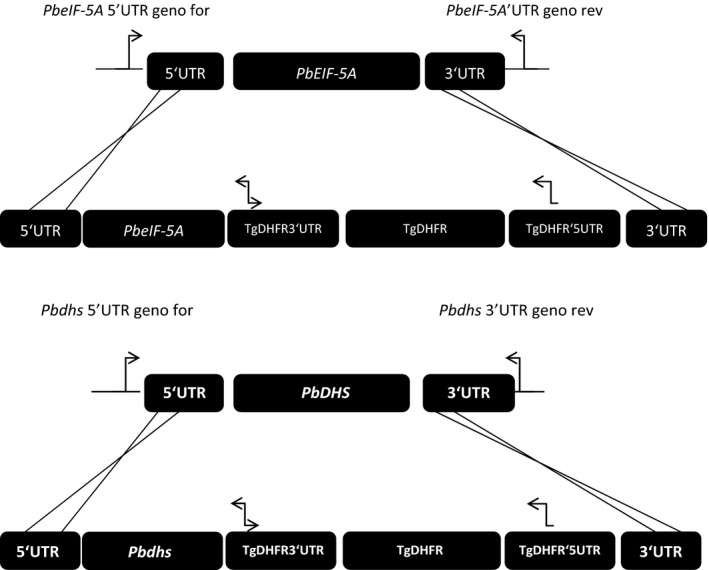
Schematic representation of the knock‐in constructs applied for a 1 : 1 substitution of the entire *eIF‐5A* (A) and *dhs* genes (B) from *Plasmodium berghei* ANKA strain. Constructs contained the coding region from either the *eIF‐5A* or *dhs* gene from *P*. *berghei* cloned in b3D+ mCherry vector behind the 5′ UTR of either gene. The full coding sequence of the *dhfr (*dihydrofolate resistance gene) with the 5′UTR and 3′ UTR is used for drug selection with pyrimethamine. Upon a double cross over event, the endogenous *eIf‐5A* or *dhs* gene from *P. berghei* is replaced by the cloned *eIF‐5A* or *dhs* genes from *P. berghei* involving the linearized 5′UTR and 3′ UTR, respectively.

**Figure 6:**
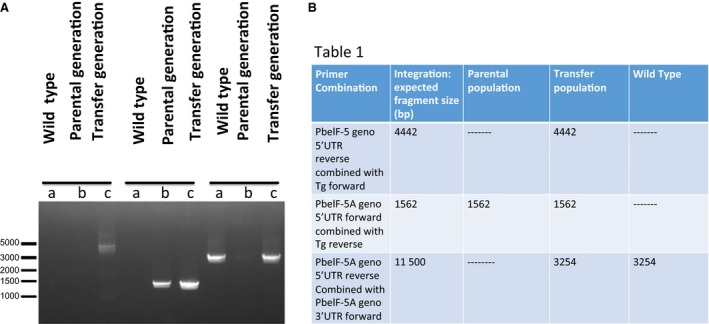
Analysis of the *Plasmodium berghei* ANKA *eIF‐5A* knock‐in after homologous recombination by double cross over. (A) PCR analysis to investigate 5′ and 3′ integration of the *eIF‐5A* gene from *P. berghei* using three different primer combinations (given in Table 1) in the transfer population (T), the parental population (P) and the wild‐type (W). (B) Table 1: 5′ integration of the *eIF‐5A* and *dhs* knock‐out parasites. Calculated fragments (bp) for the primer combination primer# genotype 5′UTR forward and primer# genotype 3′UTR rev after PCR analysis.

In order to control for gene targeting and hence to analyze whether the lack of recombination was due to the essential roles of *eIF‐5A* and *dhs* genes for parasite survival or whether other reasons prevent targeting of the *eIF‐5A* and *dhs* gene locus, we next included a knock‐in approach (Fig. 5). This approach was based on a 1 : 1 substitution of the endogenous genes from *P. berghei* ANKA strain against the cloned *eIF‐5A* and *dhs* genes from *P. berghei* in recombinant b3D*+* mCherry vector. First, parasites were transfected with the *eIF‐5A* knock‐in construct (Fig. 5A) that would result in a functional gene copy. When genotypical analysis was performed by PCR, a combination of primers *PbeIF‐5A* geno 5′UTR and *PbeIF‐5A* geno 3′UTR were applied resulting in the expected fragment of 3254 bp in the parental population and in the wild‐type (Fig. 6). However, in the transfer population a signal of 3254 bp appeared instead of the expected signal of 11 500 bp (Fig. 6, part B, Table 1) after integration suggesting that the whole fragment cannot be amplified under these conditions without an extended activity of the Taq polymerase or a long range PCR. Next, 5′ integration was tested using primer combinations *PbeIF*‐*5A* geno 5′UTR and *T. gondii* forward primer resulting in a fragment of 4442 bp in the transfer population. As expected this fragment was absent in wild‐type *P. berghei* and in the parental population. When 3′ integration was tested, the expected signal of 1562 bp was detected in both the transfer and parental population (Fig. 6, part B, Table 1).

**Figure 7:**
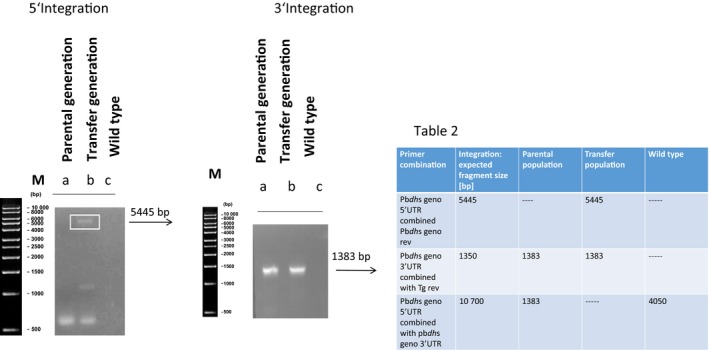
Genotypical analysis of integration after knock‐in into the *dhs* locus after gene targeting by homologous recombination. PCR analysis from three different primer combinations was employed to prove 5′ and 3′ integration of the *dhs* gene from *Plasmodium berghei*. These primer combinations are given in Table 2. Table 2: Calculated fragments (bp) of integration after knock‐in into *Plasmodium berghei dhs* locus for three different primer combinations after PCR analysis.

Next, we analyzed knock‐in mutants for the integration of the *dhs* locus (Fig. 7, Table 2). The calculated band of 5445 bp for 5′ integration applying primer combination # 5′UTR genotype forward and primer *T. gondii* reverse was only detectable in the transfer population and absent in the parental population suggesting a low transfection efficiency. It seems likely that only a small amount of transfected schizonts is present in the parental population which resulted in a very faint band monitored by PCR. As expected, the band of 5445 bp is absent in the wild‐type *P. berghei* ANKA strain. Genotypical analysis of 3′ integration with the primer combination *Pbdhs* 3′UTR genotype forward and primer *T. gondii* reverse resulted in the expected band of 1383 bp in the parental and transfer population and in the absence of the wild‐type (Fig. 7, Table 2). A complete integration applying the primer combination *Pbdhs* forward geno 5′UTR and *Pbdhs* geno 3′UTR reverse with an expected band of 10 700 bp could not be detected in the parental and transfer population as observed already for the *eIF‐5A* locus due to the size of the amplificate and the limited capacity of the Taq polymerase. In sum, our data show that both genes can be integrated into the *P. berghei* ANKA strain genome.

## Discussion

It was recently shown that hypusine modification in eukaryotic initiation factor 5A is emerging as a crucial regulatory principle in infectious diseases, inflammatory diseases like diabetes and cancer 32. Prolin‐repeat rich proteins, which are targets of eIF‐5A, are involved in connected protein‐protein interaction networks. Scaffold proteins which increase the assembly of these protein complexes in such networks and essential proteins under hypusine‐dependent‐translational control (hubs) might be responsible for the lethal phenotype in multicellular organisms after depletion of the hypusine biosynthetic genes 33. Based on these findings, the question was pursued whether either the *eIF‐5A* or the *dhs* gene is essential for intra‐erythrocytic proliferation of *Plasmodium* parasites. To our knowledge, we here describe the first experiments which demonstrate that a knock‐out of both *eIF‐5A* and *dhs* genes in *Plasmodium* by targeted gene disruption in the rodent malaria parasite *P. berghei* is not possible.

Although gene disruption in *Plasmodium* by homologous recombination has provided important insights into gene function, genomic integration has been hampered by low transfection and recombination efficiencies and the propensity of this parasite to maintain the episomal replicating plasmid. Our results clearly demonstrate that transgenic knock‐out parasites for either *eIF‐5A* or *dhs* genes did not occur in a uniform manner, but instead led to a mixed population of parasites that continued to replicate the plasmids episomically (episomal replicants) (Figs 4 and 6). The failure to prove either 3′ or 5′ integration in the transgenic *eIF‐5A* or *dhs* knock‐out parasites further supports this notion although two different, independent transfections were performed. In case of the transgenic *dhs* knock‐out parasite (Fig. 4B), a lower transfection efficiency than the expected one i.e. 10^−3^ to 10^−4^ might have enhanced episomal expression. Unsuccessful knock‐out studies were also reported for *P. falciparum* lipoic acid protein ligase A 34 which is indispensable for parasite growth in the erythrocytic stages. The authors were not able to clone out a mutant line PfLpLA1 in *P. falciparum* after two independent transfection experiments and three drug selection cycles. However, no integration into the parasite genome could be detected. These results were attributed to the indespensible role of the LpLA1 protein in the erythrocytic stages. In a second approach the authors circumvented the problem by double transfection with a parasitic line containing the KO LpL∆A1 plasmid already and the *P. berghei* LpLAORF which was 70% homologous to the *P. falciparum* gene and under control of a *P. falciparum* promotor. This allowed continuous expression of the gene throughout the erythrocytic stages episomically without recombination and a knock‐out of the KO LpL∆A1 plasmid 34.

One technical improvement might be to use a rapid genetic integration method into *P. berghei* utilizing mycobacteriophage Bxb1 serine integrase which provides a greater genetic and phenotypic homogeneity within transgenic lines 35. Moreover, piggyBAC transposases and zinc‐finger nucleases could also be applied 36. Alternatively, PlasmoGEM vectors could be employed 37. However, hitherto targeting constructs for the *eIF5a* and *dhs* genes are not available in the PlasmoGEM vector collection. A third, technical improvement might be the application of novel genome editing techniques based on the RNA‐guided CRISPR (clustered regularly interspaced short palindromic repeats‐ the nuclease Cas (CRISPR‐associated proteins) system. This technique has now been reported for *P. falciparum* 38, 39, providing a powerful new approach that can be used to interrogate the malaria parasite genome. The CRISPR/Cas system has the advantage to introduce a double strand‐break (DSB) at a specific site on a chromosome which can be repaired by homologous recombination since the error‐prone nonhomologous endjoining (NHEJ) pathway is absent in *Plasmodium*. This technique has been successfully applied for gene deletion, knock‐in, and allelic replacement in the *P. yoelii* genome 39.

Since full disruption of the two genes of interest was not successful, hence to control for gene targeting at the desired gene loci we next included a knock‐in strategy. 5′ integration and 3′ integration was shown in both cases for the *eIF‐5A* and *dhs* genes demonstrating that both genes are accessible for gene targeting (Figs 6 and 7A,B).

It would be of further interest to investigate a 1 : 1 substitution of the *dhs* gene of *P. berghei* with the *dhs* gene from *P. vivax* since both genes share only 70% identity on the amino acid level. Over recent years, successful knock‐ins were employed for the construction of animal disease models for pharmacological testing. In this context, the human p53 tumor suppressor gene was applied for a knock‐in in a mouse model for carcinogenic testing 40. This could be an important issue for further pharmacological intervention of the plasmodial hypusine pathway in a rodent model.

## Conclusion

Our results demonstrate that the *dhs* and *eIF‐5A* genes might be essential for parasitic intra‐erythrocytic proliferation. Given the crucial function for either the *eIF‐5A* or *dhs* gene for pathological blood‐stage progression after this initial reverse genetic approach, a further alternative technique in molecular genetic systems in *Plasmodium* 41 is indeed necessary to define their essential role(s) in the process of malarial infection. Recently, barcoded, genetic modification vectors containing sequences from the *P. berghei* genome with high efficiency for integration enable reverse genetic screening in one inbred mouse which can be phenotyped by next generation sequencing 41. Hence, it was shown in PlasmoDB that transcript levels of *eIF‐5A* and *dhs* genes are significantly increased in rings and trophozoites while considerable transcript levels can only be observed for the *eIF‐5A* gene in ookinetes. Conditional mutants might aid in addressing this question. It has been recently reported that disruption of the *dhs* gene in mice leads to a severe defect in hematopoiesis and spleen due to reduced hypusine modification 29 while depletion of the *dohh* gene is attributed to liver necrosis and inflammation. Since the malaria infection starts in the liver before the erythrocytic stages are involved it would be of considerable interest to investigate the impact of a disrupted hypusine modification system on a biochemical basis in these developmental stages.

## Author contributions

AK, DK, and MK proposed the scientific hypothesis and organized the study. DK, MK, and JMS performed the experiments. DK, MK, JMS, AKM, and AK analyzed and interpreted the data. DK, MK, and AK wrote the paper. All authors discussed the results and commented on the manuscript. AK was responsible for financial support.
